# Effects of *Torulaspora delbrueckii* and *Saccharomyces cerevisiae* Co-Fermentation on the Physicochemical and Flavor Compounds of Huaniu Apple Cider

**DOI:** 10.3390/molecules29081750

**Published:** 2024-04-12

**Authors:** Chaozhen Zeng, Yuwen Mu, Jing Yuan, Haiyan Zhang, Juan Song, Sanjiang Kang

**Affiliations:** Agricultural Product Storge and Processing Research Institute, Gansu Academy of Agricultural Sciences, Lanzhou 730070, China; zengchaozhen@gsagr.cn (C.Z.); muyw@gsagr.cn (Y.M.); yuanjing@gsagr.cn (J.Y.); zhanghaiyan@gsagr.cn (H.Z.); songjuan@gsagr.cn (J.S.)

**Keywords:** Huaniu apple, *Torulaspora delbrueckii*, mixed fermentation, Huaniu apple cider, quality analysis

## Abstract

The effects of different fermentation methods utilizing *Torulaspora delbrueckii* 1004 and *Saccharomyces cerevisiae* 32169 on the physicochemical properties, organic acid content, polyphenol and flavonoid concentrations, antioxidant activity, and volatile aroma compounds of Huaniu apple cider were investigated in this study. Employing methods of single inoculation, co-inoculation, and sequential inoculation, it was found that sequential fermentation exhibited strong fermentative power in the initial stages, effectively reducing the content of soluble solids and achieving a balanced composition of malic, succinic, and citric acids while maintaining a lower titratable acidity. Sequential inoculation was observed to significantly enhance the total polyphenols and flavonoids, as well as the antioxidant capacity (*p* < 0.05). Specifically, in the synthesis of volatile aroma compounds, sequential inoculation significantly enhanced the richness and diversity of the Huaniu apple cider’s aromas, particularly in terms of the concentration of ester compounds (*p* < 0.05). Principal component analysis further confirmed the superiority of sequential inoculation in terms of aroma component diversity and richness. The findings of this study suggest that sequential inoculation of fermentation with non-*Saccharomyces* and *S. cerevisiae* is an effective strategy for optimizing the flavor characteristics of Huaniu apple cider, offering valuable theoretical support and practical guidance for enhancing cider quality and fostering the development of new products.

## 1. Introduction

The Huaniu apple, as one of China’s national geographical indication products, enjoys a prestigious reputation in both domestic and international markets and is one of the top three apple brands worldwide [1,2]. Originating from Huanuizhai in Maiji District, Tianshui City, Gansu Province, China, the Huaniu apple has been cultivated since its introduction in 1956 and has become representative of the Marshal series of fine varieties developed in Tianshui City [3]. The Huaniu apple is widely loved for its well-shaped fruit, bright color, sweet taste, and unique aroma [4]. Its fruit is rich in nutrients, including sugars, organic acids, various vitamins, and minerals [5], making it an ideal raw material for fermentation.

Cider is a beverage derived from the partial or full alcoholic fermentation of apple juice, constituting an indispensable product within the apple industry [6]. In contemporary cider production, selected wine yeasts are inoculated into the juice to enhance the efficiency of alcoholic fermentation, thereby ensuring the fermentation process’s controllability and reproducibility, as well as the predictability of the sensory quality of the fermented drinks [7]. However, this approach’s limitation is the relative uniformity and lack of diversity in the resulting cider flavors [8]. The use of mixed fermentation with *Saccharomyces* and non-*Saccharomyces* was found to increase the complexity of aromas by augmenting the types and quantities of volatile aroma components, which significantly impacted the flavor formation of the fermented product [9,10,11]. *Torulaspora delbrueckii* is a non-*Saccharomyces* that was found to positively impact the chemical characteristics of fruit wines when used in sequential or simultaneous fermentation with *Saccharomyces cerevisiae* [12,13]. Mixed fermentation with *T. delbrueckii* and *S. cerevisiae* significantly affects the content of key volatile compounds, such as 2-phenylethanol, isoamyl acetate, fatty acid esters, C4-C10 fatty acids, and vinyl phenols, in both dry and sweet styles of wines [14]. Beyond the analysis of aroma components in wine, *T. delbrueckii* positively influences phenolic compounds related to sensory attributes in white wines, leading to increased concentrations and astringency, as well as enhanced mouthfeel [15]. *T. delbrueckii* has also been applied in the primary fermentation of sparkling wines, pure secondary fermentation, and mixed secondary fermentations, increasing glycerol concentration, reducing volatile acidity, improving effervescence and foam stability, and further enhancing the flavor complexity of sparkling wines during secondary fermentation [16,17]. Beyond its positive impact on the sensory quality of wine, *T. delbrueckii* has also been applied to the fermentation of durian wine, raspberry wine, strawberry wine, mead, and cashew wine. Co-fermentation with lactic acid bacteria, involving both alcoholic and lactic acid fermentation, significantly increased the content of higher alcohols (isoamyl alcohol, active amyl alcohol, isobutanol, and 2-phenylethanol), ethyl acetates (ethyl acetate), and esters (ethyl caprate and ethyl laurate) in durian wine [18]; volatile esters, ketones, and terpenes were abundant in raspberry wine, while other aroma intensities were relatively lower, and different fermentation modes imparted “fruity”, “sweet”, “floral”, and “spicy” sensory characteristics to raspberry wine [19]; and *T. delbrueckii* significantly affected the color and aroma components of strawberry wine under different mixed culture modes [20]. Mead made with *T. delbrueckii* had a low alcohol content, high residual sugar, and retained the main aroma of honey [21]; the fermentation of cashew wine produced volatile compounds, such as phenylethanol, 2-phenylethyl acetate, and 3-methyl-1-pentanol, which are beneficial to the product’s sensory performance [22]. Despite these studies, research investigating the performance of *T. delbrueckii* in Huaniu apple cider has not yet been conducted.

The aim of this study was to investigate the impact of different fermentation methods using *S. cerevisiae* and *T. delbrueckii*, both simultaneous and sequential inoculation, on the physicochemical properties, organic acids, total polyphenols and flavonoids, and volatile aroma compounds of Huaniu apple cider. This work can assist winemakers in optimizing fermentation strategies by leveraging *T. delbrueckii* to enhance the sensory quality of Huaniu apple cider.

## 2. Results and Discussion

### 2.1. Effects of Different Inoculation Methods on the Physicochemical Properties of Huaniu Apple Cider

**Figure 1:**
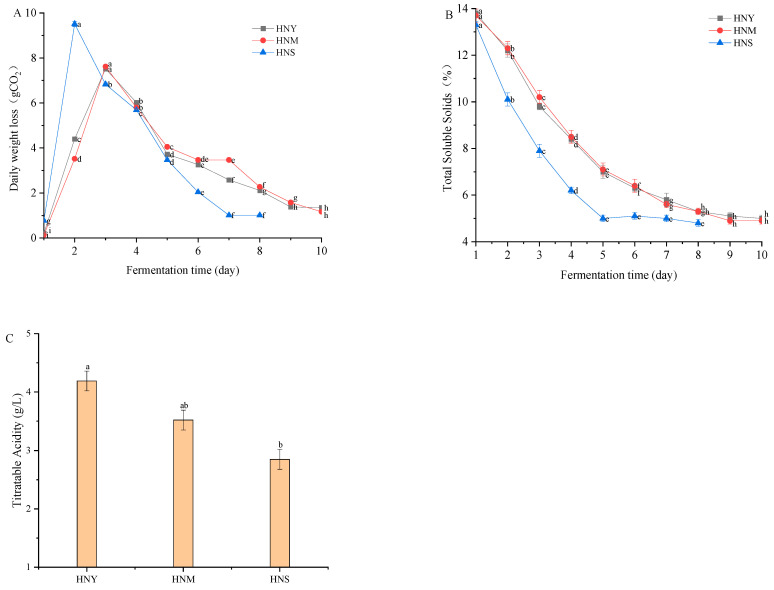
Changes in fermentative activity (**A**), soluble solids content (**B**), and titratable acidity (**C**) of Huaniu apple cider. Note: HNY: fermentation with a single strain of 10^5^ cfu/mL CICC 32169; HNM: mixed fermentation with 10^5^ cfu/mL CICC 32169 and 10^6^ cfu/mL CICC 1004; HNS: sequential fermentation with 10^5^ cfu/mL CICC 32169, followed by 10^6^ cfu/mL CICC 1004. Different lowercase letters denote statistically significant differences (*p* < 0.05).

Figure 1A illustrates the changes in yeast alcohol fermentation power during the fermentation of Huaniu apple cider with complex microbial cultures. As can be observed from the figure, all treatments showed a significant increase on the second day (*p* = 0.0001), with HNS (sequential fermentation with 10^5^ cfu/mL CICC 32169 followed by 10^6^ cfu/mL CICC 1004) exhibiting the greatest increase. HNY (fermentation with a single strain of 10^5^ cfu/mL CICC 32169) and HNM (mixed fermentation with 10^5^ cfu/mL CICC 32169 and 10^6^ cfu/mL CICC 1004) reached their peak on the third day, followed by a continuous decline; HNS decreased rapidly after the second day, then stabilized at a lower level. From the fifth day onwards, the rate of decrease in HNY and HNM slowed down, whereas the decline in HNS significantly slowed after the sixth day (*p* = 0.0001). These changes suggest that HNY and HNM may have entered a more gradual reaction phase in the later stages of the experiment, while HNS rapidly reached a steady state in the early stages of the experiment. The changes in soluble solids content during the fermentation of Huaniu apple cider with complex microbial cultures are shown in Figure 1B. The soluble solids content in the HNY-treated cider decreased from 13.8% on the first day to 5.0% on the tenth day, a total reduction of 63.8%. For the HNM-treated cider, the soluble solids content slightly dropped from 13.7% on the first day to 12.3% on the second day, then steadily decreased to 4.9% on the tenth day, totaling a 64.2% reduction. Meanwhile, the soluble solids content in the HNS-treated cider decreased from 13.3% on the first day to 10.1% on the second day, a more significant initial decrease compared to the other treatments (*p* = 0.0001). This content continued to decrease in this treatment, reaching 4.8% on the tenth day, a total reduction of 63.9%. These results indicate that all treatment methods showed a declining trend in soluble solids content over time. The decrease in soluble solids content for HNY and HNM was similar, both exhibiting a relatively steady gradual decline. The trend for HNS was steeper in the first two days, but from the third day onward, the rate of decrease became more gradual, aligning with the trends of the other treatments. Over the entire 10-day fermentation period, the soluble solids content in all three treatments reduced by approximately two-thirds, indicating similar efficiency among the three methods. However, the more rapid decrease in soluble solids content at the start of the HNS treatment may suggest greater initial effectiveness. This effect could be due to differences in dissolution rates under different treatment conditions or varying impacts of the methods on the stability of soluble solids. Ultimately, by the tenth day, the soluble solids content in all three treatments was nearly the same, which might indicate that, regardless of initial efficiency, the final steady-state effects were similar. The results of the titratable acidity content in Huaniu apple cider fermented with complex microbial cultures are presented in Figure 1C. The results show that the titratable acidity content of the cider treated with HNY was 4.19 g/L, that for the HNM treatment it was 3.52 g/L, and that for the HNS treatment it was 2.85 g/L. The HNY treatment had the highest titratable acidity content, which was significantly higher than that of the HNS treatment (*p* = 0.0261), but the difference was not significant compared to the HNM treatment (*p* = 0.1083). The titratable acidity content of the HNM treatment was lower than that of HNY, but it was not statistically significantly higher than that of HNS, nor significantly lower than that of HNY (*p* = 0.1083). The HNS treatment had the lowest titratable acidity content, which was significantly lower than that of the HNY treatment (*p* = 0.0261). If the goal is to reduce the content of titratable acidity, the HNS treatment might be the most effective method of fermentation.

### 2.2. The Impact of Different Inoculation Methods on the Organic Acid Content in Huaniu Apple Cider

The effects of compound microbial fermentation on the organic acid content in Huaniu apple cider are shown in Table 1. Malic acid was identified as the most abundant organic acid in Huaniu apple cider, followed by succinic and citric acids. In HNY fermentation, the malic acid content was the highest at 2934.68 mg/L, while citric and succinic acid concentrations were lower, at 115.98 mg/L and 730.50 mg/L, respectively. The concentrations of acetic acid, fumaric acid, and propionic acid were moderate. Under HNM fermentation, the concentrations of citric and fumaric acids were higher, being 197.03 mg/L and 11.28 mg/L, respectively. The acetic acid concentration was also higher, reaching 139.34 mg/L, while malic and succinic acids were present at moderate levels. In HNS fermentation, malic and succinic acid concentrations were higher, at 1919.86 mg/L and 789.03 mg/L, respectively; the citric acid concentration was also high, at 195.31 mg/L, and the acetic acid concentration was the lowest. Malic acid provides a strong and sharp taste, but an excessively high concentration may result in a harsh flavor [23]. Citric acid is important for the flavor and acid balance of fruit wine; an appropriate level of citric acid can enhance the aroma of the wine, but too high a concentration might lead to an overly tart taste [24]. Succinic acid plays a role in the acidity regulation of the wine, but an excessive amount may negatively impact the flavor [25]. A higher concentration of acetic acid might cause the wine to have an unpleasant vinegar taste [26]. Fumaric and propionic acids are generally present in lower quantities in fruit wines and have a lesser impact on the overall flavor. In summary, sequential fermentation with HNS seems to provide a better balance, enhancing the concentrations of malic and succinic acids while maintaining a higher level of citric acid and reducing the risk of acetic acid. This could lead to a better flavor balance and overall quality of the wine.

### 2.3. Analysis of Polyphenol and Flavonoid Contents in Huaniu Apple Cider Prepared via Different Inoculation Methods

**Figure 2:**
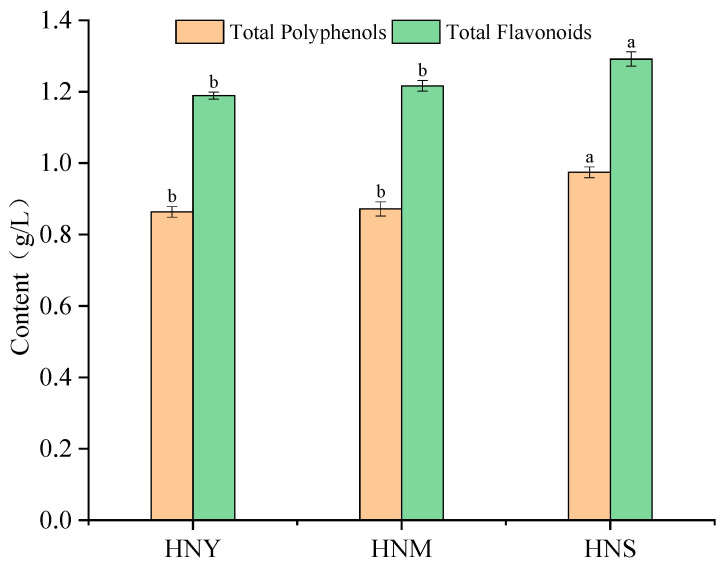
The contents of polyphenols and flavonoids in Huaniu apple cider fermented with different inoculation methods. Values in columns of the same pattern with different superscript letters are significantly different (*p* < 0.05).

Total polyphenols and flavonoids are important components in fruit wine, influencing its antioxidant properties, color, and flavor. Generally, a high content of these compounds is associated with a better quality of fruit wine. The impact of different inoculation methods on the polyphenol and flavonoid contents in Huaniu apple cider is illustrated in Figure 2. In the HNY fermentation of Huaniu apple cider, the contents of total polyphenols and flavonoids were relatively low, at 0.86 g/L and 1.22 g/L, respectively. In the HNM fermented cider, there was an increase of 1.16% in total polyphenols and 2.46% in flavonoids. The HNS fermentation showed the highest increase in both total polyphenols and flavonoids, with increments of 12.79% and 5.74%, respectively. From these data, it can be concluded that the sequential fermentation HNS method appears to be most favorable for enhancing the content of total polyphenols and flavonoids in the cider, potentially indicating superior antioxidant properties and health benefits of the cider fermented by this method. The mixed fermentation HNM also showed improvements over the single-strain fermentation HNY, but these were not as significant as the improvements observed for the sequential fermentation HNS in terms of polyphenols (*p* = 0.0313) and flavonoids (*p* = 0.0406). Previous research indicates that phenolic compounds possess a variety of biological activities, particularly antioxidative properties, which are beneficial for health [27]. All tested wines contained a substantial amount of phenolic substances, suggesting a high level of antioxidant activity. Therefore, for producing fruit wine with higher contents of total polyphenols and flavonoids, sequential fermentation with HNS is identified as the optimal fermentation method.

### 2.4. The Impact of Different Inoculation Methods on the Antioxidant Activity of Huaniu Apple Cider

**Figure 3:**
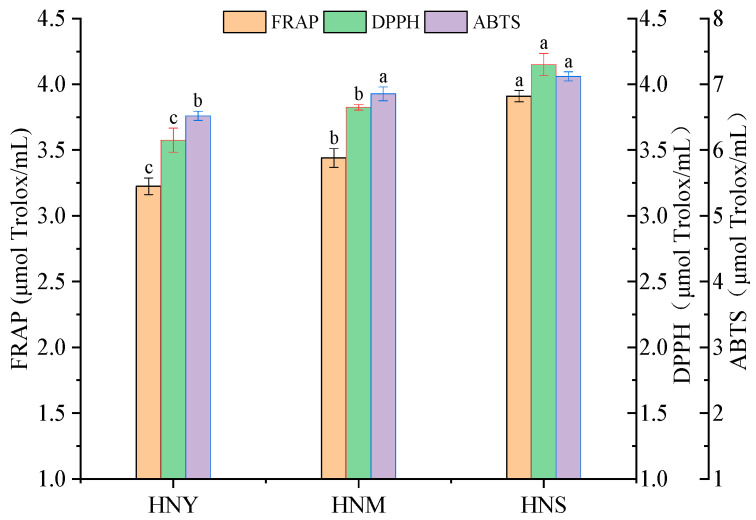
Antioxidant activity of Huaniu apple cider fermented with different inoculation methods. Values in columns of the same pattern with different superscript letters are significantly different (*p* < 0.05).

The impact of different inoculation methods on the antioxidant activity of Huaniu apple cider was assessed using FRAP, DPPH, and ABTS assays (Figure 3). Mixed fermentation improved the values of FRAP, DPPH, and ABTS to varying degrees. The FRAP value for the HNM treatment increased by approximately 6.83%, the DPPH value by about 6.70%, and the ABTS value by about 5.06%. For the HNS treatment, the increases were approximately 21.43% in FRAP, 15.92% in DPPH, and 9.20% in ABTS. Compared to the control group, HNY, mixed microbial fermentation (HNM and HNS) enhanced the antioxidant capacity of Huaniu apple cider, with the increase for HNS being more pronounced than for HNM. Specifically, the FRAP and DPPH assays showed significant increases (*p* = 0.0032; *p* = 0.0099), while the ABTS assay also showed an increase, though to a lesser extent (*p* = 0.0131). This suggests that mixed microbial fermentation may positively affect the antioxidant components in Huaniu apple cider, thereby enhancing its antioxidant capacity. In particular, the HNS treatment demonstrated the largest increase in all three assays, indicating its potential effectiveness in enhancing antioxidative properties. The analysis suggests that the reason for this may be the extracellular metabolites secreted by non-*Saccharomyces* yeasts during the early stages of fermentation (potentially including certain proteinaceous substances, as well as alcohol, aldehyde, and ketone compounds), which affect the fermentation of *Saccharomyces* yeasts. This, in turn, could be conducive to the formation and accumulation of antioxidative substances, thereby enhancing the capacity to scavenge free radicals [28].

### 2.5. The Effect of Different Inoculation Methods on the Volatile Aroma Compounds in Huaniu Apple Cider

**Figure 4:**
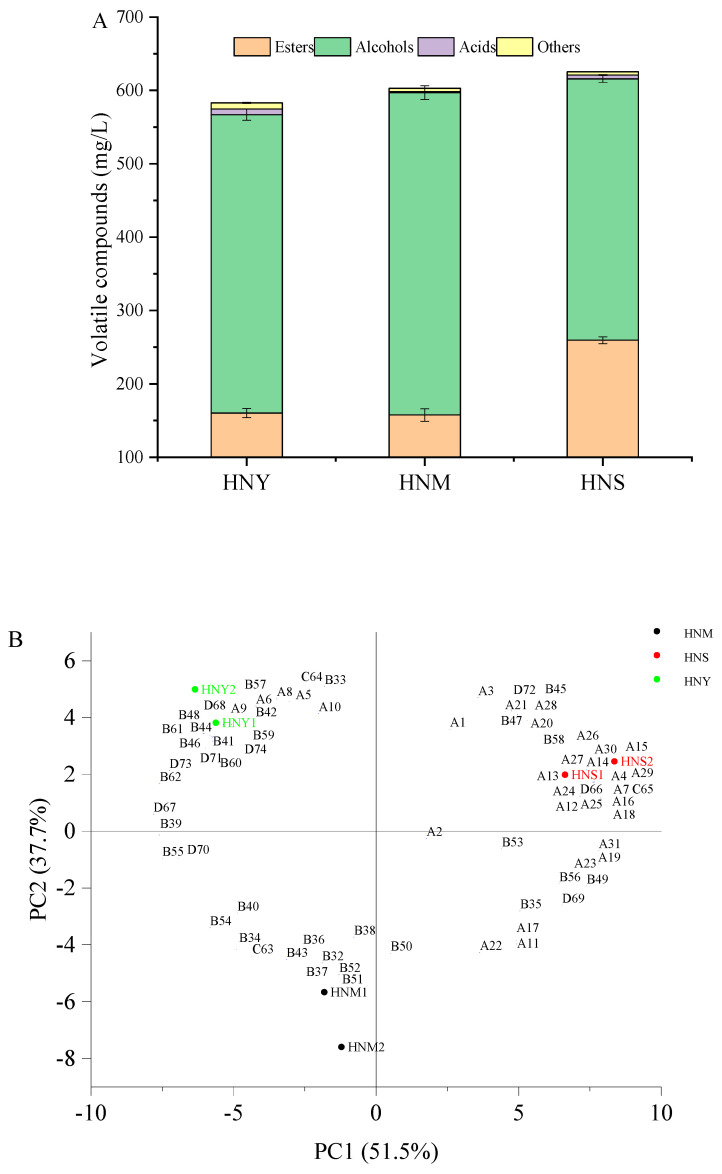

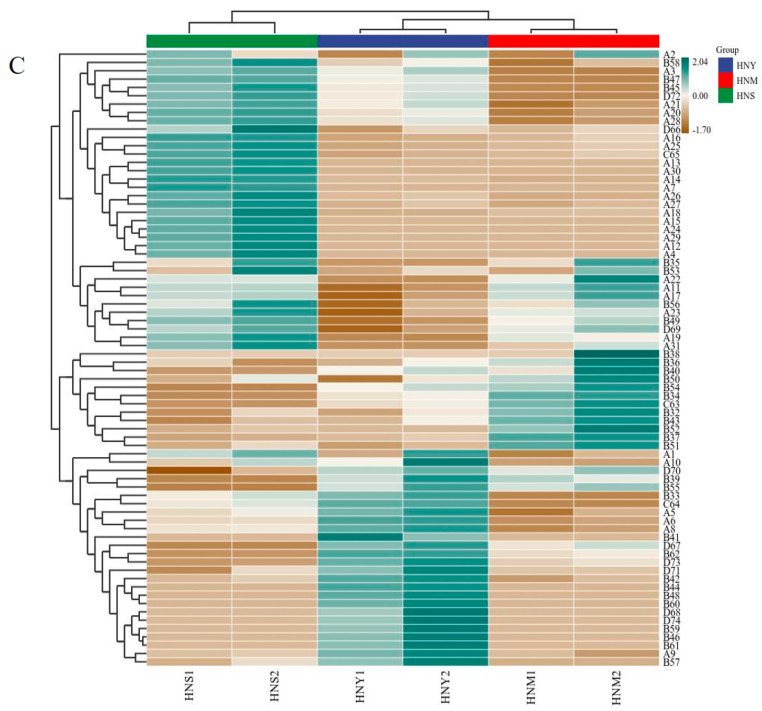
Total aroma compound content (**A**), principal component analysis (**B**), and cluster heatmap (**C**) of volatile compounds of Huaniu apple cider produced by pure and mixed culture fermentations.

**Figure A1:**
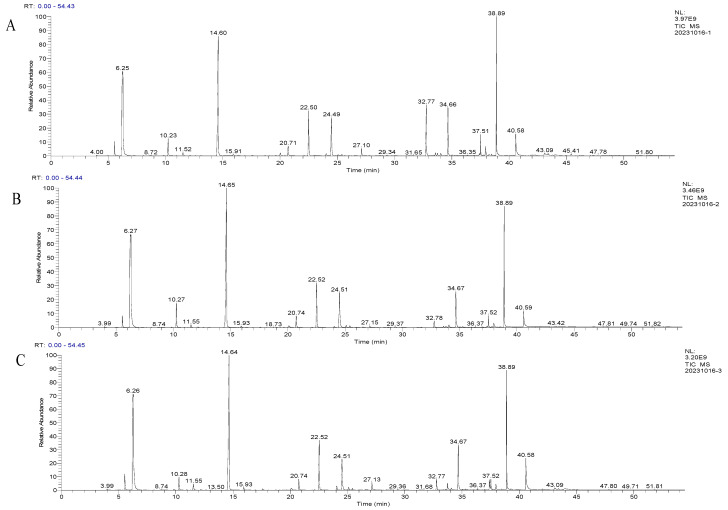
GC-MS total ion chromatogram of aromatic components in Huaniu apple cider produced by HNY (**A**), HNM (**B**), and HNS (**C**).

Fermentation aroma is a major component of the aroma profile in fruit wines, and the impact of volatile compounds on the flavor of Huaniu apple cider primarily depends on their actual concentrations in the cider. Volatile compounds in Huaniu apple cider fermented according to different methods were analyzed using HS-SPME-GC-MS, with the results and the gas chromatography chromatograms presented in Table 2 and Figure A1. Table 2 shows the variations in the content of esters, alcohols, acids, and other compounds across the three fermentation methods. A total of 74 volatile compounds were identified in Huaniu apple cider fermented through three different methods, categorized into esters, higher alcohols, volatile fatty acids, and other compounds. In the HNY, HNM, and HNS fermentations, 62, 52, and 59 volatile compounds were detected, respectively. As shown in Figure 4A, there were significant differences in the impact of different treatments on the esters (*p* = 0.0009), alcohols (*p* = 0.0325), acids (*p* = 0.0029), and other compounds (*p* = 0.0083) in Huaniu apple cider. The HNS treatment exhibited the highest content of aroma substances at 625.40 mg/L, while the control (HNY) had the lowest at 583.07 mg/L. The total volatile compound content in both HNM and HNS samples was higher than in HNY, indicating that mixed microbial fermentation can positively contribute to the flavor diversity of Huaniu apple cider.

Esters are major contributors to the characteristic aroma components of fruit wines, primarily formed through the esterification of acids and alcohols during the fermentation process. When ester compounds reach a certain concentration, it has been observed that they can increase the complexity of a wine’s aroma, imparting composite scents such as floral and fruity notes. Table 2 reveals that a total of 31 ester compounds were identified in Huaniu apple cider, including 4 acetate esters (ethyl acetate, ethyl 3-methyl-1-butanolate, ethyl 5-hexen-1-olate, and ethyl 2-phenylacetate), 14 ethyl esters of fatty acids, and 13 other esters. Acetate esters are formed by the condensation of higher alcohols with acetyl-CoA and are catalyzed in yeast cells by alcohol acyltransferase (AAT) genes ATF1 and ATF2 [29]. The results in Table 2 show that the total content of acetate esters in the HNS fermentation samples was the highest, at 23.90 mg/L, with the concentration of ethyl 2-phenylacetate (A25) in the HNS samples being significantly higher than in the other two fermentation samples (*p* = 0.0015). Among the four acetate esters, the content of ethyl 2-phenylacetate(A25) accounted for over 49% of the total acetate esters in each sample, greatly contributing to the enhancement of rose and honey flavors in Huaniu apple cider [30]. In Huaniu apple cider, the contents of ethyl acetate (A1) and ethyl 5-hexen-1-olate (A10) showed no significant differences. In terms of ethyl esters, ethyl hexanoate (A8), ethyl octanoate (A11), ethyl decanoate (A14), ethyl benzoate (A17), ethyl 9-decenoate (A20), and ethyl laurate (A26) were present in higher concentrations in all Huaniu apple cider samples. Comparing the ethyl ester contents in HNS, HNM, and HNY samples, we identified some significant advantages of the HNS fermentation method. Regarding the production of ethyl decanoate (A14), its concentration was considerably higher in HNS fermented Huaniu apple cider, reaching 75.21 mg/L, than in the HNM (8.92 mg/L) and HNY (13.01 mg/L) samples (*p* = 0.0001). This suggests that the HNS fermentation process is more favorable for the production of ethyl decanoate. Moreover, the concentrations of ethyl octanoate (A11) and ethyl 9-decenoate (A20) in HNS fermented Huaniu apple cider were 63.15 mg/L and 60.01 mg/L, respectively, both higher than in the HNY fermented cider (ethyl octanoate at 51.16 mg/L and ethyl 9-decenoate at 53.92 mg/L) and comparable to the concentrations in HNM fermented cider (ethyl octanoate at 65.73 mg/L and ethyl 9-decenoate at 48.85 mg/L). The 13 other types of esters were relatively low in all Huaniu apple cider samples. Overall, compared to fermentation with *S. cerevisiae* alone, the involvement of non-*Saccharomyces* yeast significantly enhances the complexity of ester components (*p* = 0.0009). The highest concentration of aroma substances in HNS fermented Huaniu apple cider demonstrates that *T. delbrueckii* can significantly increase the content of ester compounds in Huaniu apple cider (*p* = 0.0009).

Higher alcohols, formed as reaction products of corresponding amino acids through the Ehrlich pathway [31], are among the most abundant volatile compounds in fermented wines. Generally, it has been observed that higher alcohols, serving as precursors to pleasant ester compounds, can enhance the complexity of a wine’s flavor when their total mass concentration is below 300 mg/L [7]. However, when their total content exceeds 400 mg/L, they may impart a strong pungent odor and cause unpleasurable effects, such as headaches, nausea, and vomiting [32]. In this study, after quantitatively analyzing the total content of higher alcohols in cider produced according to three different fermentation methods, the results showed that the highest total content of higher alcohols was found for the HNM fermentation method, at 439.26 mg/L, while in the HNY and HNS fermentations, the total contents were 406.77 mg/L and 356.17 mg/L, respectively. The common higher alcohols with higher contents in all Huaniu apple ciders included 2-methyl-1-propanol, 3-methyl-1-butanol, 1-hexanol, 1-heptanol, 6-methyl-5-hepten-2-ol, and phenylethanol. 3-methyl-1-butanol was highly concentrated in all fermentations, with the highest concentration in HNM (281.91 mg/L). Phenylethanol had its highest concentration in the HNY fermentation (125.68 mg/L), while being relatively lower in HNM (101.52 mg/L) and HNS (86.83 mg/L); 2-methyl-1-propanol was most abundant in the HNM fermentation (29.09 mg/L), followed by HNY (20.89 mg/L), and was lowest in HNS (14.14 mg/L), a trend similar to 3-methyl-1-butanol; 1-hexanol’s concentration varied significantly across the fermentation methods, with an increase in HNM (16.31 mg/L) (*p* = 0.0304); 6-methyl-5-hepten-2-ol’s content showed minor variation across the methods, being slightly higher in HNM (2.39 mg/L); and 1-heptanol was somewhat higher in the HNS fermentation (3.56 mg/L). Compared to pure *S. cerevisiae* fermentation, co-inoculation fermentation with *T. delbrueckii* and *S. cerevisiae* can increase the content of higher alcohols by 7.99%. It is speculated that this could be due to the autolysis of non-*Saccharomyces* in the later stages of fermentation, providing nutrients to *S. cerevisiae*. Alternatively, non-*Saccharomyces* may possess certain enzymatic activities that provide a nutritional source for *S. cerevisiae* [33]. However, sequential inoculation fermentation with *T. delbrueckii* followed by *S. cerevisiae* led to a 12.44% decrease in the content of higher alcohols in Huaniu apple cider. This reduction was not only related to the quantity of yeasts in the fermentation system but also potentially due to the conversion of higher alcohols into ester compounds by non-*Saccharomyces* [34]. Some studies have also shown that the content of higher alcohols decreases in sequential fermentation with *T. delbrueckii* compared to single fermentation with *S. cerevisiae* [35,36]. It is believed that the reduction in all these higher alcohols is achieved through fermentation initiated by individual non-*Saccharomyces* strains [37]. Higher alcohols have a strong and pungent odor, significantly affecting the taste and quality of fruit wine. Both high and low concentrations of higher alcohols can adversely affect the flavor of wine. Insufficient amounts can lead to a bland taste, while excessive amounts can create a spicy, foul, and unpleasantly bitter flavor. Additionally, it was noted that an excessive content of higher alcohols can have anesthetic effects on the human body or cause intoxication in consumers. Therefore, it is necessary to reduce the content of higher alcohols in fruit wines to improve their flavor.

Volatile fatty acids are important volatile compounds contributing to the complexity and balance of aromas in fruit wines [24]. However, when the total mass concentration of volatile fatty acids is below 20 mg/L, it has been found that they can impart a rich fruitiness to wine [38]. In this study, three volatile acid compounds were detected in Huaniu apple cider, including capric acid, isobutyric acid, and 9-decenoic acid. The total content of these compounds in the ciders fermented by different methods ranged from 1.23 to 7.57 mg/L, all below 20 mg/L. Among these, capric acid was found in a high concentration in the cider produced according to the HNY method, at 6.87 mg/L; isobutyric acid was highest in the HNM method, at 0.62 mg/L; and 9-decenoic acid was most abundant in the HNS method, at 1.74 mg/L. Capric acid is a residual fragment of long-chain fatty acids needed for yeast cell membrane synthesis during fermentation; its accumulation is toxic to yeast growth and imparts an unpleasant odor to wine [39]. This experiment found a significant decrease in capric acid content in Huaniu apple cider during sequential inoculation fermentation with *T. delbrueckii* and *S. cerevisiae* (*p* = 0.0245). Some studies have also indicated that capric acid is characteristic of *S. cerevisiae* fermentation, giving wine a slightly putrid odor. The content of n-capric acid decreases after mixed fermentation [40]. Our results are consistent with these findings.

In addition to the main esters, higher alcohols, and volatile fatty acid compounds, certain aldehydes, ketones, and terpenes also influence the aroma of fruit wines. In Huaniu apple cider, seven ketone compounds, two aldehyde compounds, and one terpene compound were detected. The total content of these substances in HNY fermented Huaniu apple cider was higher than in mixed microbial fermented cider. In the HNS mixed microbial fermented Huaniu apple cider, the content of *β*-damascenone was significantly higher than in HNY mixed microbial fermented cider (*p* = 0.0331). *β*-Damascenone is characterized by baked apple, floral, and honey aromas [41], and this metabolite has a very low odor threshold, at 0.05 micrograms per liter, meaning that even minor changes can significantly impact the sensory evaluation of wine [29].

A principal component analysis (PCA) was applied to analyze the relationship between Huaniu apple cider fermented using three different methods and their volatile compound profiles. As shown in Figure 4B, ciders fermented by the three methods were well differentiated in the PCA plot. HNS and HNY fermented Huaniu apple ciders were primarily distributed in the positive quadrant of PC1 and PC2, while HNM fermented cider was mainly located in the negative quadrant. HNS fermented cider, positioned in the first quadrant, was rich in flavor substances, including isobutyl isobutyrate(A2), *(R)*-3,7-dimethyl-6-decen-1-ol(B58), ethyl propionate(A3),3,4,5-trimethyl-4-heptanol(B47), 2-nonen-1-ol(B45), *β*-damascenone(D72), ethyl 4-decenoate(A21), ethyl 9-decenoate (A20), isobutyl 2,2,4-trimethyl-1,3-pentanediol ester(A28), 3-methylbutanal(D66), 3-methylbutyl octanoate(A16), ethyl 2-phenylacetate(A25), 9-decenoic acid(C65), isobutyl decanoate(A13), ethyl 10-undecenoate(A30), ethyl decanoate(A14), amyl valerate(A7), ethyl laurate(A26), decyl 3-methylbutyrate(A27), methyl cis-9-decenoate(A18), ethyl undecylenate (A15), methyl 4-hydroxybutyrate(A24), ethyl 3-hydroxydecanoate(A29), hexyl isovalerate(A12), and ethyl 2,3-epoxybutyrate(A4). HNY fermented cider, located in the second quadrant, mainly comprised ethyl 5-hexen-1-olate(A10), dihydro-2-methyl-3(2H)-thiophenone(D70), 4-methyl-1-pentanol(B39), 1-nonanol(B55), 2-heptanol(B33), decanoic acid(C64), butyl butyrate(A5), ethyl 3-methyl-1-butanolate(A6), ethyl hexanoate(A8), 3-methyl-1,5-pentanediol(B41), 3-octanone(D67), phenethyl alcohol(B62), 2,5-dimethylbenzaldehyde(D73), 2,4,4-trimethyl-3-(3-methylbutyl)cyclohex-2-enone(D71), 3-methyl-1-pentanol(B42), 3-Hexen-1-ol(B44),2,3-dimethyl-3-octanol(B48), 2-methyl-1-decanol(B60), 1-decen-3-one(D68), 6,10-dimethyl-5,9-undecadien-2-one(D74), 7-methyl-3-methylene-6-decen-1-ol(B59), (*E*)-2-decen-1-ol(B46), benzyl alcohol(B61), hexyl hexanoate(A9), and 1-decanol(B57). HNM fermented cider, situated in the third quadrant, included 1-pentanol(B38), 1-butanol(B36), (*S*)-2-heptanol(B40), 6-methyl-5-hepten-2-ol(B50), (*E*)-2-octen-1-ol(B54), 2-methyl-1-propanol(B34), isobutyric acid(C63), 1-propanol(B32), 1-hexanol(B43), 2-nonanol(B52), 3-methyl-1-butanol(B37), and 2-ethyl-1-hexanol(B51). To visually display the relationship between different fermentation methods for Huaniu apple cider and volatile compounds, a heatmap and hierarchical cluster analysis (HCA) were used, as shown in Figure 4C. The clear color differences in the cluster analysis indicate significant variations in the content of volatile aroma compounds in Huaniu apple cider fermented by different methods. According to the heatmap, HNS fermented cider forms one group, characterized by a greater variety and quantity of ester compounds; HNM and HNY fermented ciders form another group, with a higher variety and quantity of alcohol compounds. These results are consistent with those of the principal component analysis.

## 3. Materials and Methods

### 3.1. Materials

Huaniu apples were obtained from Maiji District (Tianshui, China). *S. cerevisiae* CICC 32169 and *T. delbrueckii* CICC 1004 were provided by the China Center of Industrial Culture Collection (CICC, Beijing, China). Yeast Extract Peptone Dextrose (YPD) Agar Medium was obtained from Beijing Land Bridge Technology Co., Ltd. (Beijing, China). All analytical or high-performance liquid chromatography (HPLC)-grade chemical and biochemical reagents were provided by Sinopharm Chemical Reagent Co., Ltd. (Shanghai, China) or Sigma Aldrich (Beijing, China).

### 3.2. Strain Activation

The activation of *S. cerevisiae* and *T. delbrueckii* was carried out with reference to the method of Zeng et al. [42], with slight modifications. Yeast was retrieved from the preserved slant culture medium utilizing an inoculating loop, with 2–3 loopfuls of the culture being inoculated into a 250 mL Erlenmeyer flask containing 100 mL of YPD (Yeast Extract Peptone Dextrose) liquid medium. This culture was incubated at 28 °C for 48 h. Post-incubation, the yeast concentrations were quantified using a hemocytometer, and the cultures were subsequently stored at 4 °C for later use.

### 3.3. Huaniu Apple Cider Fermentation

Huaniu apple juice was prepared by crushing and juicing Huaniu apples, and 350 mL of the juice was dispensed into a 500 mL Erlenmeyer flask. The juice was then inoculated with *T.delbrueckii* (CICC 1004) and *S. cerevisiae* (CICC 32169) under three different cultivation regimes: a monoculture with *S. cerevisiae* at 10^5^ cfu/mL (HNY), a co-culture combining *S. cerevisiae* at 10^5^ cfu/mL and *T.delbrueckii* at 10^6^ cfu/mL (HNM), and a sequential culture where *S. cerevisiae* at 10^5^ cfu/mL was initially inoculated, followed by *T. delbrueckii* at 10^6^ cfu/mL (HNS). The fermentation process was conducted at a constant temperature of 20 °C to establish both monoculture and mixed culture systems. Samples were systematically collected from each fermentation batch every 24 h, with triplicates for each sampling point, and were preserved at −80 °C pending further analysis.

### 3.4. Measurement of Yeast Ethanol Fermentation Capacity, Soluble Solids Content, and Titratable Acidity

Yeast ethanol fermentation capacity: The determination was conducted following the method described by Zeng et al. [43]. Utilizing the gravimetric approach, the extent of yeast’s fermentation capacity was quantified based on the mass loss attributed to the volatilization of carbon dioxide. The mass was measured every 24 h until the completion of fermentation. Soluble solids content: The measurement was performed using a digital refractometer. Titratable acidity: The measurement was conducted in accordance with GB/T 15038-2006, “General Analysis Methods for Wine and Fruit Wine” [44].

### 3.5. Organic Acid Analysis

The determination of organic acid was carried out by slightly optimizing a previously reported method [45]. Samples of 2 mL were centrifuged at 10,000 r/min for 5 min, and the supernatant was filtered by a 0.45 μm filter membrane. The column type was the Waters Atlantis C18 column (250 mm × 4.5 mm, 5 μm), the column temperature was 30 °C, the mobile phase was 0.05 mmol/L H_3_PO_4_/methanol = 95:5 (*v*/*v*), the flow rate was 0.8 mL/min, and the detection wavelength was 210 nm. The sample was filtered through a membrane pore of 0.45 μm, and the injection volume was 10 μL.

### 3.6. Phenolic Compound Analysis

#### 3.6.1. Determination of Total Phenolic Content (TPC)

The total phenolic content (TPC) was determined utilizing the Folin–Ciocalteu method [46]. A quantity of 0.5 mL of apple cider was mixed with 0.5 mL of Folin–Ciocalteu reagent and 1 mL of 20% Na_2_CO_3_. The mixture was then incubated at room temperature for 10 min in the dark, and the volume was adjusted to 5 mL with distilled water. After resting at room temperature for 30 min, the absorbance was analyzed and monitored at 760 nm using a SPECTR Amax 190 microplate reader (Molecular Devices Corp., Sunnyvale, CA, USA). A calibration curve was prepared using gallic acid as a reference compound, and the results were expressed as milligrams per milliliter of gallic acid equivalents (GAEs).

#### 3.6.2. Determination of Total Flavonoid Content (TFC)

Total flavonoid content (TFC) was determined using the aluminum chloride colorimetric method [47]. Quantities of 2.5 mL of 70% ethanol solution and 0.15 mL of 5% NaNO_2_ were added to 0.5 mL of the sample. The mixture was shaken and allowed to stand for 6 min. Then, 0.3 mL of 10% AlCl_3_ solution was added to the mixture and left to stand for 5 min. Finally, 1 mL of 1 mol/mL NaOH was added to the mixture, and the volume was adjusted to 5 mL with 70% ethanol solution. The mixture was oscillated and left to stand for 10 min. The absorbance was read at 510 nm using a spectrophotometer. The total flavonoid content was determined using a standard curve prepared with rutin. TFC was expressed as the weight of rutin per milliliter of the sample (mg).

### 3.7. Antioxidant Analysis

#### 3.7.1. DPPH· Radical Scavenging Activity

The DPPH· free radical method was carried out according to the method of Loganayaki et al., with modifications [48]. A quantity of 20 µL of the supernatant was added to 380 µL of DPPH· solution (0.1 mM). The tubes were allowed to stand for 20 min at 27 °C. Changes in the absorbance of the samples were measured at 517 nm. The antioxidant efficiency was determined as the time when the concentration of substrate caused a 50% loss in absorbance, and the results were depicted as Trolox equivalents.

#### 3.7.2. ABTS Free-Radical Scavenging Assay

This assay was carried out by slightly optimizing a previously reported method [49]. Briefly, 3.3 mg of sodium persulfate and 19.4 mg of ABTS^+^ were added to an aluminum-wrapped amber with 5 mL of distilled water, mixed well, and left for 16 h in the dark. The reagents were prepared by mixing anhydrous ethanol (1:10 dilution), 190 mL diluted ABTS^+^, and 10 mL sample for 20 min. The absorbance was taken by a SPECTRA max190 microplate reader at 734 nm, and the results were depicted as Trolox equivalents.

#### 3.7.3. Ferric-Ion-Reducing Antioxidant Power (FRAP)

The FRAP analysis was conducted by slightly optimizing a previously reported method [50]. The reagent comprised 25 mL of acetate buffer solution (0.3 M, pH 3.6), 2.5 mL of TPTZ (0.01 M) in 40 mmol/L HCl, and 2.5 mL of FeCl_3_ (0.02 M), which were mixed, shaken, and warmed at 37 °C for 30 min. Then, 190 mL of these reagents was added to 10 mL of the sample at 37 °C for 20 min, and its absorbance was measured by a SPECTRA max 190 microplate reader at 593 nm. One milliliter of the sample produced Fe^2+^-TPTZ/min, a unit of enzyme activity. The total antioxidant capacity was depicted as Trolox equivalents.

### 3.8. Volatile Composition Analysis

#### 3.8.1. Extraction of Cider Aroma Components

Headspace solid-phase micro-extraction (SPME) was performed by taking 5 mL of the sample and adding 1 g of NaCl and 50 μL of the internal standard 3-octanol (63.08 mg/L) into a 15 mL headspace vial, vortexing and mixing the vials, and refrigerating them overnight. The SPME system (TriPlus RSH Autosampler-SPME, Thermo Fisher Scientific, Waltham, MA, USA) used a 50/30 μm DVB/CAR/PDMS extraction head. The extraction conditions included an adsorption phase at 60 °C for 30 min, followed by a 5 min holding time.

#### 3.8.2. Chromatographic and Mass Spectrometric Conditions

GC conditions: The inlet temperature was set at 250 °C, and He was used as the carrier gas with a flow rate of 1.2 mL/min. The injection volume was 1 μL, using the splitless mode. The chromatographic column was a DB-WAX column (30 m × 0.25 mm × 0.25 μm). The heating procedure involved maintaining a constant temperature of 40 °C for 3 min, followed by an increase to 180 °C at a rate of 6 °C/min for 2 min, and finally raising the temperature to 230 °C at a rate of 10 °C/min for 6 min. MS conditions: EI ion source used with an electron energy of 70 eV, an ion source temperature of 200 °C, and an interface temperature of 230 °C. The scan range was set from 33.00 to 450.00 amu.

### 3.9. Statistical Analysis

All experiments were conducted in triplicate, and data analysis was performed using Microsoft Excel 2016 and SPSS 24.0 software. Graphical processing was carried out with Origin 2021 software. The data were subjected to a one-way analysis of variance (ANOVA), and the significance of the differences between means was determined by Tukey’s multiple range test (*p* < 0.05).

## 4. Conclusions

The effects of different fermentation methods involving *T. delbrueckii* and *S. cerevisiae* on the physicochemical properties, organic acid content, polyphenol and flavonoid levels, antioxidant activity, and volatile aroma compounds of Huaniu apple cider were thoroughly investigated in this study. It was demonstrated that sequential inoculation fermentation significantly improved the physicochemical components of the Huaniu apple cider, especially in terms of enhancing the antioxidant capacity and aroma characteristics. Sequential inoculation not only facilitated effective alcoholic fermentation but also regulated the content of organic acids and increased the levels of polyphenols and flavonoids, thereby boosting the antioxidant capacity of the Huaniu apple cider. Most notably, this method substantially increased the volatile aroma compounds of the cider, particularly ester compounds, providing richer and more diverse aroma compounds. These findings not only offer new insights into the application of mixed fermentation technology in fruit wine production but also provide a scientific basis for the flavor enhancement and innovation of fruit wine products such as Huaniu apple cider. However, further exploration is needed before the commercial application of mixed fermentation technology, including optimizing fermentation conditions and assessing the potential of different non-*Saccharomyces* strains. Future research may focus on gaining a deeper understanding of the impact of mixed fermentation on the microbial ecology and aroma formation mechanisms of apple cider, as well as exploring the potential value of other non-*Saccharomyces*.

## Figures and Tables

**Table 1:** Organic acid contents of Huaniu apple cider prepared with different inoculation methods (mg/L).

Organic Acid		Content (mg/L)	
	HNY	HNM	HNS
Malic Acid	2934.68 ± 6.35 ^a^	1468.13 ± 7.99 ^c^	1919.86 ± 5.11 ^b^
Citric Acid	115.98 ± 2.50 ^b^	197.03 ± 1.61 ^a^	195.31 ± 2.05 ^a^
Succinic Acid	730.50 ± 2.56 ^b^	666.96 ± 2.97 ^c^	789.03 ± 2.05 ^a^
Acetic Acid	117.12 ± 2.61 ^b^	139.34 ± 1.56 ^a^	73.39 ± 2.47 ^c^
Fumaric Acid	6.90 ± 0.48 ^b^	11.28 ± 0.59 ^a^	11.53 ± 0.82 ^a^
Propionic Acid	224.76 ± 2.96 ^c^	439.31 ± 2.50 ^a^	261.09 ± 2.42 ^b^

Note: Values in the same row with different superscript letters are significantly different (*p* < 0.05).

**Table 2:** Concentration of aroma compounds in Huaniu apple cider fermented with pure and mixed cultures (mg/L).

No.	^a^ RI	Compound	^b^ Concentration (mg/L)			^c^ ID
			HNY	HNM	HNS	
A1	872	ethyl acetate	0.17 ± 0.10 ^a^	0.09 ± 0.03 ^a^	0.20 ± 0.03 ^a^	RI/MS
A2	1094	isobutyl isobutyrate	0.16 ± 0.07 ^a^	0.17 ± 0.08 ^a^	0.19 ± 0.04 ^a^	RI/MS
A3	998	ethyl propionate	0.47 ± 0.1 ^a^	nd	0.65 ± 0.07 ^a^	RI/MS
A4	1051	ethyl 2,3-epoxybutyrate	nd	nd	0.06 ± 0.01 ^a^	RI/MS
A5	1220	butyl butyrate	0.94 ± 0.07 ^a^	0.47 ± 0.10 ^b^	0.67 ± 0.06 ^b^	RI/MS
A6	1223	ethyl 3-methyl-1-butanolate	9.03 ± 0.17 ^a^	4.44 ± 0.33 ^c^	5.83 ± 0.06 ^b^	RI/MS
A7	1225	amyl valerate	nd	nd	0.02 ± 0.00 ^a^	RI/MS
A8	1232	ethyl hexanoate	4.26 ± 0.21 ^a^	2.08 ± 0.18 ^c^	2.94 ± 0.04 ^b^	RI/MS
A9	1603	hexyl hexanoate	2.2 ± 0.35 ^a^	0.76 ± 0.18 ^b^	0.95 ± 0.06 ^b^	RI/MS
A10	1294	ethyl 5-hexen-1-olate	0.05 ± 0.03 ^a^	0.01 ± 0.00 ^a^	0.03 ± 0.01 ^a^	RI/MS
A11	1440	ethyl octanoate	51.16 ± 2.09 ^b^	65.73 ± 4.24 ^a^	63.15 ± 0.3 ^a^	RI/MS
A12	1425	hexyl isovalerate	nd	nd	0.26 ± 0.06 ^a^	RI/MS
A13	1750	isobutyl decanoate	nd	nd	1.38 ± 0.1 ^a^	RI/MS
A14	1648	ethyl decanoate	13.01 ± 0.41 ^b^	8.92 ± 0.64 ^c^	75.21 ± 0.35 ^a^	RI/MS
A15	1651	ethyl undecylenate	nd	nd	0.08 ± 0.01 ^a^	RI/MS
A16	1670	3-methylbutyl octanoate	1.20 ± 0.14 ^b^	1.68 ± 0.31 ^b^	4.58 ± 0.10 ^a^	RI/MS
A17	1644	ethyl benzoate	3.6 ± 0.20 ^b^	4.5 ± 0.28 ^a^	4.35 ± 0.04 ^a^	RI/MS
A18	1667	methyl cis-9-decenoate	nd	0.01 ± 0.00 ^b^	0.11 ± 0.03 ^a^	RI/MS
A19	1699	ethyl cis-2-decenoate	nd	0.12 ± 0.08 ^b^	0.22 ± 0.04 ^a^	RI/MS
A20	1703	ethyl 9-decenoate	53.92 ± 1.15 ^b^	48.85 ± 1.23 ^c^	60.01 ± 0.88 ^a^	RI/MS
A21	1705	ethyl 4-decenoate	0.78 ± 0.14 ^a^	0.20 ± 0.14 ^b^	1.17 ± 0.11 ^a^	RI/MS
A22	2565	benzyl benzoate	nd	0.15 ± 0.08 ^a^	0.10 ± 0.00 ^a^	RI/MS
A23	1778	ethyl phenylacetate	0.43 ± 0.07 ^b^	0.58 ± 0.01 ^ab^	0.66 ± 0.07 ^a^	RI/MS
A24	1361	methyl 4-hydroxybutyrate	nd	nd	0.33 ± 0.06 ^a^	RI/MS
A25	1779	ethyl 2-phenylacetate	8.99 ± 0.34 ^b^	10.07 ± 0.55 ^b^	17.84 ± 0.89 ^a^	RI/MS
A26	1856	ethyl laurate	8.69 ± 0.21 ^b^	8.14 ± 0.1 ^b^	13.81 ± 0.98 ^a^	RI/MS
A27	1921	decyl 3-methylbutyrate	0.30 ± 0.14 ^b^	0.21 ± 0.13 ^b^	2.02 ± 0.18 ^a^	MS
A28	1932	isobutyl 2,2,4-trimethyl-1,3-pentanediol ester	0.82 ± 0.11 ^b^	0.34 ± 0.08 ^c^	1.31 ± 0.08 ^a^	MS
A29	1967	ethyl 3-hydroxydecanoate	nd	nd	0.34 ± 0.06 ^a^	MS
A30	2082	ethyl 10-undecenoate	nd	nd	0.62 ± 0.06 ^a^	RI/MS
A31	2198	hexyl 2-phenylacetate	nd	0.17 ± 0.10 ^b^	0.39 ± 0.08 ^a^	RI/MS
B32	1037	1-propanol	0.13 ± 0.03 ^ab^	0.22 ± 0.06 ^a^	0.12 ± 0.06 ^b^	RI/MS
B33	1327	2-heptanol	0.12 ± 0.01 ^a^	nd	0.07 ± 0.01 ^b^	RI/MS
B34	1036	2-methyl-1-propanol	20.89 ± 1.02 ^b^	29.09 ± 1.20 ^a^	14.14 ± 0.18 ^c^	RI/MS
B35	1251	2-pentanol	0.02 ± 0.00 ^b^	0.04 ± 0.01 ^a^	0.04 ± 0.01 ^a^	RI/MS
B36	1125	1-butanol	0.68 ± 0.03 ^a^	0.76 ± 0.06 ^a^	0.66 ± 0.03 ^a^	RI/MS
B37	1202	3-methyl-1-butanol	232.62 ± 3.51 ^b^	281.91 ± 4.62 ^a^	224.93 ± 2.53 ^b^	RI/MS
B38	1251	1-pentanol	0.1 ± 0.00 ^a^	0.13 ± 0.04 ^a^	0.1 ± 0.00 ^a^	RI/MS
B39	1294	4-methyl-1-pentanol	0.11 ± 0.04 ^a^	0.08 ± 0.01 ^a^	nd	RI/MS
B40	1464	(*S*)-2-heptanol	0.06 ± 0.01 ^a^	0.09 ± 0.07 ^a^	nd	RI/MS
B41	1320	3-methyl-1,5-pentanediol	0.04 ± 0.01 ^a^	nd	nd	RI/MS
B42	1330	3-methyl-1-pentanol	0.54 ± 0.04 ^a^	0.19 ± 0.04 ^b^	0.23 ± 0.03 ^b^	RI/MS
B43	1361	1-hexanol	14.48 ± 0.51 ^b^	16.31 ± 0.40 ^a^	13.85 ± 0.54 ^b^	RI/MS
B44	1333	3-hexen-1-ol	0.4 ± 0.04 ^a^	nd	nd	RI/MS
B45	1379	2-nonen-1-ol	0.13 ± 0.03 ^b^	nd	0.24 ± 0.04 ^a^	RI/MS
B46	1822	(*E*)-2-decen-1-ol	0.04 ± 0.01 ^a^	nd	nd	RI/MS
B47	1827	3,4,5-trimethyl-4-heptanol	3.44 ± 0.24 ^b^	nd	5.95 ± 0.23 ^a^	MS
B48	1841	2,3-dimethyl-3-octanol	0.08 ± 0.01 ^a^	nd	nd	RI/MS
B49	1465	1-heptanol	1.69 ± 0.23 ^c^	2.94 ± 0.27 ^b^	3.56 ± 0.21 ^a^	RI/MS
B50	1454	6-methyl-5-hepten-2-ol	2.15 ± 0.11 ^a^	2.39 ± 0.11 ^a^	2.21 ± 0.08 ^a^	RI/MS
B51	1435	2-ethyl-1-hexanol	0.52 ± 0.03 ^b^	0.78 ± 0.03 ^a^	0.55 ± 0.04 ^b^	RI/MS
B52	1524	2-nonanol	0.11 ± 0.00 ^b^	0.25 ± 0.06 ^a^	0.11 ± 0.01 ^b^	RI/MS
B53	1541	linalool	0.20 ± 0.01 ^a^	0.22 ± 0.04 ^a^	0.24 ± 0.06 ^a^	RI/MS
B54	1620	(*E*)-2-octen-1-ol	0.22 ± 0.04 ^a^	0.35 ± 0.10 ^a^	nd	RI/MS
B55	1672	1-nonanol	0.28 ± 0.10 ^a^	0.24 ± 0.04 ^a^	nd	RI/MS
B56	1714	3-(methylthio)-1-propanol	0.67 ± 0.10 ^c^	0.89 ± 0.11 ^b^	0.98 ± 0.14 ^a^	RI/MS
B57	1771	1-decanol	0.27 ± 0.03 ^a^	0.19 ± 0.00 ^b^	0.20 ± 0.01 ^b^	RI/MS
B58	1779	(*R*)-3,7-dimethyl-6-decen-1-ol	0.85 ± 0.07 ^b^	0.67 ± 0.13 ^c^	1.16 ± 0.1 ^a^	MS
B59	1786	7-methyl-3-methylene-6-decen-1-ol	0.07 ± 0.03 ^a^	nd	nd	MS
B60	1802	2-methyl-1-decanol	0.06 ± 0.01 ^a^	nd	nd	RI/MS
B61	1880	benzyl alcohol	0.12 ± 0.04 ^a^	nd	nd	RI/MS
B62	1915	phenylethanol	125.68 ± 1.26 ^a^	101.52 ± 1.98 ^b^	86.83 ± 0.51 ^c^	RI/MS
C63	1580	isobutyric acid	0.24 ± 0.04 ^b^	0.62 ± 0.11 ^a^	nd	RI/MS
C64	2281	decanoic acid	6.87 ± 0.21 ^a^	nd	3.6 ± 0.71 ^b^	RI/MS
C65	2347	9-decenoic acid	0.46 ± 0.07 ^c^	0.61 ± 0.08 ^b^	1.74 ± 0.14 ^a^	RI/MS
D66	915	3-methylbutanal	0.1 ± 0.03 ^b^	0.11 ± 0.01 ^b^	0.21 ± 0.06 ^a^	RI/MS
D67	1241	3-octanone	0.09 ± 0.01 ^a^	0.05 ± 0.01 ^a^	nd	RI/MS
D68	1320	1-decen-3-one	0.03 ± 0.01 ^a^	nd	nd	RI/MS
D69	1425	6-methyl-5-hepten-2-one	0.21 ± 0.01 ^b^	0.26 ± 0.01 ^a^	0.27 ± 0.01 ^a^	RI/MS
D70	1505	dihydro-2-methyl-3(2H)-thiophenone	0.28 ± 0.01 ^a^	0.27 ± 0.01 ^ab^	0.21 ± 0.03 ^b^	RI/MS
D71	1681	2,4,4-trimethyl-3-(3-methylbutyl)cyclohex-2-enone	0.41 ± 0.03 ^a^	0.33 ± 0.01 ^b^	0.32 ± 0.03 ^b^	MS
D72	1814	*β*-damascenone	0.40 ± 0.02 ^b^	nd	0.79 ± 0.10 ^a^	RI/MS
D73	1830	2,5-dimethylbenzaldehyde	1.06 ± 0.07 ^a^	0.75 ± 0.03 ^b^	0.62 ± 0.01 ^b^	MS
D74	1953	6,10-dimethyl-5,9-undecadien-2-one	0.16 ± 0.07 ^a^	nd	nd	MS

Note: ^a^ RI, retention indices determined using the DB-Wax (30 m × 0.25 mm × 0.25 μm) capillary column. ^b^ nd, not detected. ^c^ Identification methods; RI: retention indices relative to C_5_–C_30_ n-alkanes; MS: detected by mass spectra (NIST 2015). In the rows, different lowercase letters represent significant differences between treatments (Tukey’s test, *p* < 0.05).

## Data Availability

Data are contained within the article.
